# Influence of bevacizumab therapy and intraretinal hemorrhage in long-term outcomes of hemorrhagic retinal arterial macroaneurysm

**DOI:** 10.1038/s41598-021-93811-7

**Published:** 2021-07-09

**Authors:** Jae Hui Kim, Jong Woo Kim, Chul Gu Kim, Young Ju Lew, Han Joo Cho

**Affiliations:** Department of Ophthalmology, Kim′s Eye Hospital, #156 Youngdeungpo-dong 4ga, Youngdeungpo-gu, Seoul, 150-034 South Korea

**Keywords:** Eye diseases, Outcomes research

## Abstract

This study aimed to evaluate the long-term visual outcomes of hemorrhagic retinal arterial macroaneurysm (RAM), particularly focusing on the influence of bevacizumab therapy and intraretinal hemorrhage (IRH) on the outcomes. This retrospective study included 49 patients diagnosed with hemorrhagic RAM. Patients were divided into the bevacizumab group and observation group depending on the whether they were administered bevacizumab treatment and the IRH group and the non-IRH group based on the presence of IRH at the fovea. Best-corrected visual acuity (BCVA) at diagnosis was compared with that at the final visit. Further, the BCVA at the final visit was compared between the study groups. Multivariate analysis was also performed to identify factors associated with poor BCVA at the final visit. The mean follow-up period was 24.8 ± 15.3 months. The mean logarithm of minimal angle of resolution BCVA was significantly improved from 1.37 ± 0.70 at diagnosis to 0.72 ± 0.62 at the final visit (P < 0.001). There was no significant difference in the BCVA at the final visit between the bevacizumab group and observation group (P = 0.576). However, the BCVA at the final visit was significantly worse in the IRH group than in the non-IRH group (P = 0.002). In multivariate analysis, the presence of IRH was significantly associated with poor BCVA (P = 0.007). Significant long-term visual improvement was noted in hemorrhagic RAM. However, the presence of IRH at the fovea was associated with poor visual prognosis. Bevacizumab therapy did not significantly influence the outcomes.

## Introduction

Retinal arterial macroaneurysm (RAM) is a disorder characterized by saccular or fusiform dilatation of the retinal artery^1^. RAM usually affects elderly females. Systemic hypertension is a well-known risk factor for RAM^2^. According to previous studies, 1 in 1500–4500 adults have RAM^3,4^.

The RAM often regresses spontaneously^5–7^. Nevertheless, various approaches, including laser photocoagulation, vitrectomy with the use of an intravitreal tissue plasminogen activator, and anti-vascular endothelial growth factor (VEGF) therapy, have been attempted to improve outcomes of this condition^2^. To date, there is no gold-standard method to treat RAM.

Hemorrhage is a frequent presentation of RAM which can lead to a sudden loss of vision^5–10^. In RAM, hemorrhage can develop at various locations—preretinal, intraretinal, or subretinal locations^5^. The visual outcomes are generally comparable between RAM with and without hemorrhage^10^. In most patients, visual acuity improves as the hemorrhage resolves^5^. However, the clinical course and visual outcomes substantially vary among patients. In some patients, visual acuity fails to recover with scarring, resulting in very poor visual outcomes^5,7,9^. In addition, the development of macular holes secondary to intraretinal hemorrhage (IRH) may impede visual recovery^11^. Moreover, dense vitreous hemorrhage requiring surgical intervention can develop due to massive bleeding from RAM^12^.

To date, numerous studies have reported the clinical course and visual outcomes of hemorrhagic RAM^5–10,13,14^. However, the sample size in these studies was relatively small. In addition, there is a paucity of data regarding long-term outcomes of anti-VEGF therapy^14,15^. Therefore, we aimed to evaluate the long-term clinical outcomes of 49 hemorrhagic RAM cases. We particularly focused on the difference in outcomes between patients who did and did not receive intravitreal bevacizumab treatment. In addition, we investigated the influence of IRH on the outcomes.

## Materials and methods

This retrospective study was conducted at Kim’s Eye Hospital. The study was approved by the Institutional Review Board (IRB) of Kim’s Eye Hospital and was conducted following the tenets of the Declaration of Helsinki. Due to the retrospective nature of this study, the need for an informed consent was waived off (Kim’s Eye Hospital IRB, Seoul, South Korea).

Patients who had been diagnosed with symptomatic RAM at Kim’s Eye Hospital between January 2012 and January 2019 were included. The inclusion criteria were as follows: (1) patients who exhibited one-disc area or greater extent of hemorrhage and (2) patients who had hemorrhage or exudation at the fovea. The exclusion criteria were as follows: (1) follow-up duration < 12 months, (2) treatment for RAM other than intravitreal bevacizumab, (3) history of vitreoretinal surgery, (4) severe media opacity that may preclude accurate imaging of the retina, and (5) > 2 months of symptom duration.

At diagnosis, ophthalmological examinations, including best-corrected visual acuity (BCVA) measurement, 90-D lens slit-lamp biomicroscopy, and fundus photography, were performed. Optical coherence tomography (OCT) and fluorescein angiography were also performed. After diagnosis, patients were treated with intravitreal bevacizumab (1.25 mg/0.05 mL of Avastin®; Genentech Inc., South San Francisco, CA) or closely observed without treatment at the discretion of the treating physician.

**Figure 1:**
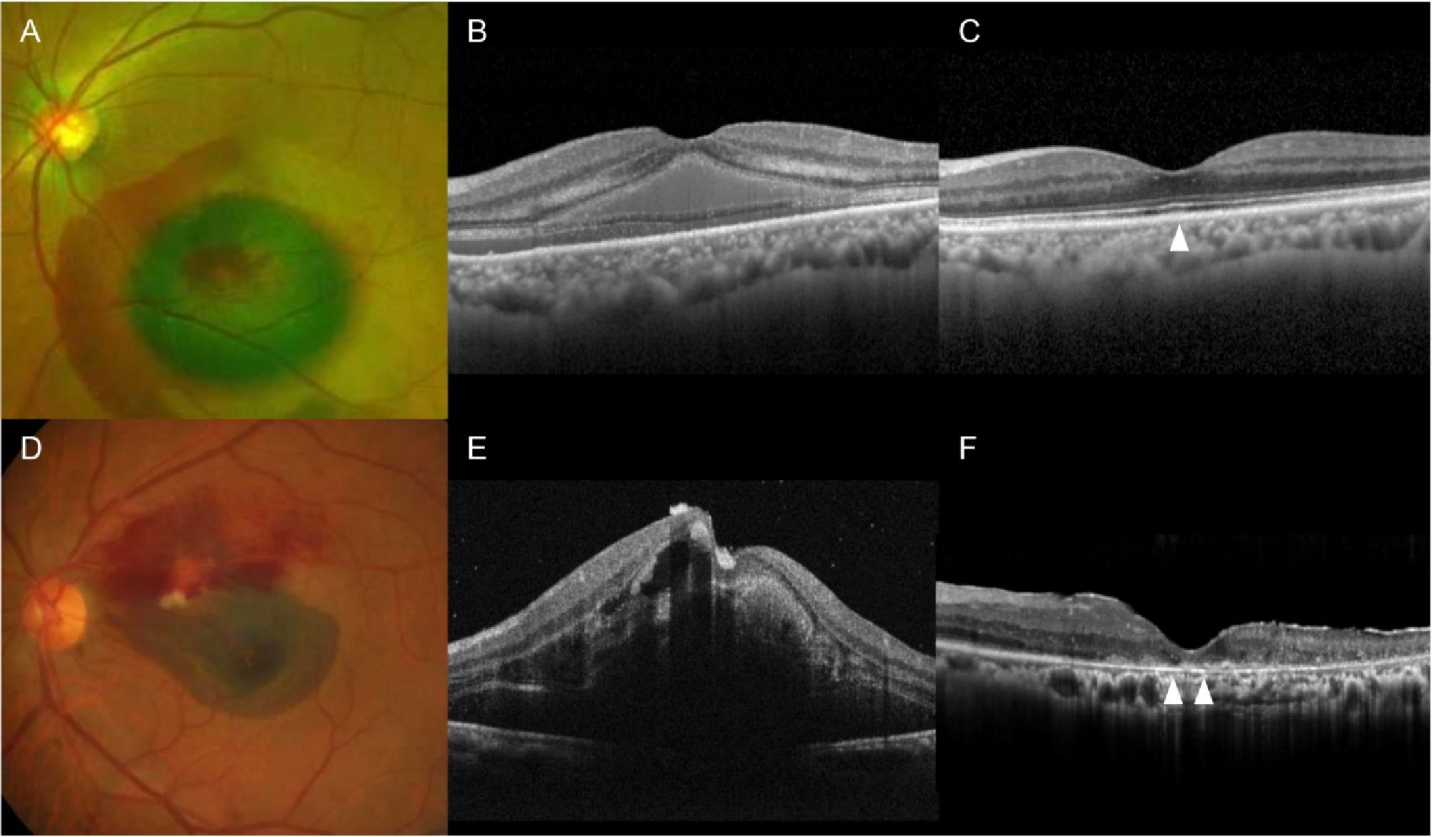
Representative cases showing difference in the status of ellipsoid zone at the final follow-up. (**A**,**B**) An 80-year-old patient was diagnosed with hemorrhagic retinal arterial macroaneurysm (RAM). (**C**) An optical coherence tomography (OCT) image taken at the final follow-up showing intact foveal ellipsoid zone (arrowhead). (**D**,**E**) A 72-year-old patient was diagnosed with RAM. (**F**) An OCT image taken at final follow-up showing disruption of foveal ellipsoid zone (arrowheads).

The BCVA values were measured using a decimal visual acuity chart and converted to logarithm of minimal angle of resolution (logMAR) values for the analysis. Counting finger and hand motion visual acuities were converted to logMAR values 2 and 3, respectively. The central foveal thickness (CFT) was defined as the vertical distance between the internal limiting membrane and Bruch’s membrane at the fovea. This was manually measured on the OCT images. When Bruch’s membrane at the fovea was not accurately identified owing to thick retinal hemorrhage, an imaginary line was drawn between the visible Bruch’s membrane line. In this case, the distance between the internal limiting membrane and this imaginary line at the fovea was defined as the CFT. Further, ellipsoid zone disruption at the fovea was identified using OCT images taken at the final follow-up (Fig. 1).

For all patients, the BCVA and CFT at diagnosis were compared to those at the final visit. In addition, the incidence of lesion reactivation and ellipsoid zone disruption was estimated. Lesion reactivation was defined as aggravation or new onset of hemorrhage or exudation and confirmed using fundus photography and OCT.

There were no strict guidelines on the use of bevacizumab in the present study. Bevacizumab use was based on the preference of the treating physician. Patients treated with intravitreal bevacizumab within 1 month of diagnosis were included in the bevacizumab group. Patients who were closely observed without any treatment and patients who received bevacizumab injection after 1 month of diagnosis were included in the observation group. The following characteristics were compared between the two groups: age, sex, presence of diabetes mellitus, presence of hypertension, use of anticoagulants, systolic and diastolic blood pressure, extent of hemorrhage, presence of foveal IRH, presence of submacular hemorrhage, and follow-up period. In addition, the BCVA and CFT at diagnosis and the final follow-up, the incidence of lesion reactivation, and the incidence of ellipsoid zone disruption were compared between the two groups.

Patients were also divided into the IRH group and the non-IRH group according to the presence of IRH at the fovea. The following characteristics were compared between the two groups: age, sex, presence of diabetes mellitus, presence of hypertension, use of anticoagulants, systolic and diastolic blood pressure, extent of hemorrhage, presence of submacular hemorrhage (i.e., subretinal hemorrhage involving the fovea), type of treatment (intravitreal bevacizumab vs. observation), and follow-up period. In addition, the BCVA and CFT at diagnosis and the final follow-up, incidence of lesion reactivation, and the incidence of ellipsoid zone disruption were compared between the two groups. In the IRH group, the incidence of fluffy hemorrhage^16^ was also identified.

To identify factors associated with BCVA at the final visit, multivariate analysis was performed. BCVA at the final visit was divided into two categories: logMAR ≥ 0.4 and logMAR < 0.4. The cutoff value logMAR 0.4 was arbitrarily determined based on the author’s clinical experience. The following factors were included in the analysis: age, sex, presence of diabetes mellitus, presence of hypertension, use of anticoagulants, systolic and diastolic blood pressure, extent of hemorrhage, presence of IRH or submacular hemorrhage, follow-up period, and type of treatment (intravitreal bevacizumab vs. observation). Due to a close association between the presence of IRH and submacular hemorrhage, the association of these factors with the visual outcome was separately analyzed: IRH was included as a factor in one multivariate analysis and submacular hemorrhage in another one.

The BCVA at diagnosis and that at the final follow-up were compared between the two groups. Comparisons of the BCVA at diagnosis and the final follow-up between (1) the bevacizumab group and the observation group and (2) the IRH group and the non-IRH group were additionally performed using the alternative logMAR values for counting finger and hand motion visual acuity: counting finger = logMAR values 2, hand motion = logMAR values 2.3. Patients were analyzed further according to their follow-up period: a long follow-up group with a follow-up period ≥ 20 months, and a short follow-up group with a follow-up period < 20 months.

Statistical analyses were performed using SPSS (version 12.0 for Windows; IBM Corporation, Armonk, NY, USA). The values were compared at different time points using a paired t-test. Comparisons between study groups were performed using the Mann–Whitney *U* test with or without Bonferroni correction, Fisher’s exact test, and the chi-square test. The association of factors with the final visual outcome was analyzed using binary logistic regression. P-values < 0.05 were considered statistically significant.

## Results

Forty-nine patients (49 eyes) were included in the study (Table 1).

**Table 1:** Characteristics of the included patients (n = 49).

Characteristics	
Age, years	78.0 ± 8.2
Male:female	12 (24.5%):37 (75.5%)
Diabetes mellitus	3 (6.1%)
Hypertension	33 (67.3%)
Use of anticoagulants	12 (24.5%)
**Blood pressure, mmHg**	
Systolic	139.0 ± 15.7
Diastolic	76.1 ± 11.4
Extent of hemorrhage, disc areas	9.0 ± 5.2
**Intraretinal hemorrhage**^**a**^	
Presence	19 (38.8%)
Absence	27 (55.1%)
Undeterminable	3 (6.1%)
**Submacular hemorrhage**	
Presence	28 (57.1%)
Absence	21 (42.9%)
**Treatment**	
Intravitreal bevacizumab	33 (67.3%)
Observation	16 (32.7%)

Data are presented as mean ± standard deviation or number (%) where applicable.

^a^Intraretinal hemorrhage is noted when it involves the fovea.

Among the patients, 12 were males and 37 were females. The mean patient age was 78.0 ± 8.2 years, and the mean follow-up period was 24.8 ± 15.3 months. During the follow-up period, cataract surgery was performed in four patients. Thirty-three patients (67.3%) were initially treated with intravitreal bevacizumab. In these patients, mean 1.9 ± 0.9 injections were administered during the follow-up period. Among the remaining 16 patients (32.7%) who did not receive any treatment initially, intravitreal bevacizumab was administered to one patient during the follow-up because of lesion reactivation.

In all 49 patients, the mean logMAR BCVA was 1.37 ± 0.70 at diagnosis, 0.82 ± 0.67 at 6 months, and 0.72 ± 0.62 at the final follow-up. BCVA at the final follow-up was significantly better than that at baseline (P < 0.001). During the follow-up period, three lines or greater improvement in BCVA was noted in 39 (79.6%) patients, whereas three lines or greater deterioration in BCVA was noted in one (2.0%) patient. The BCVA remained stable in the other nine patients (18.4%). The mean CFT was 673.5 ± 263.9 µm at diagnosis, 232.5 ± 75.7 µm at 6 months, and 228.4 ± 99.5 µm at the final follow-up. The CFT at the final follow-up was significantly lower than that at baseline (P < 0.001). During the follow-up period, lesion reactivation was noted in six patients (12.2%), of which five patients developed reactivation within 6 months of diagnosis.

Thirty-three patients (67.3%) were included in the bevacizumab group, and 16 patients (32.7%) were included in the observation group. Table 2 shows comparisons of characteristics between the two groups.

**Table 2:** Comparisons of characteristics between the bevacizumab group and the observation group.

Characteristics	Bevacizumab group (n = 33)	Observation group (n = 16)	P-value
Age, years	77.2 ± 8.6	79.7 ± 7.5	0.601^†^
Male:female	9 (27.3%):24 (72.7%)	3 (18.8%):13 (81.3%)	0.726^‡^
Diabetes mellitus	2 (6.1%)	1 (6.3%)	1.000^‡^
Hypertension	26 (78.8%)	7 (43.8%)	0.023^††^
Use of anticoagulants	9 (27.3%)	3 (18.8%)	0.726^‡^
**Blood pressure, mmHg**			
Systolic	139.1 ± 15.0	138.9 ± 17.9	0.847^†^
Diastolic	76.1 ± 11.4	79.7 ± 14.7	0.171^†^
Extent of hemorrhage, disc areas	7.9 ± 4.9	11.2 ± 5.4	0.039^†^
Presence of intraretinal hemorrhage^a^	13 (40.6%)	6 (42.9%)	1.000^††^
Presence of submacular hemorrhage	16 (48.5%)	12 (75.0%)	0.079^††^
Follow-up period, months	25.4 ± 16.2	23.4 ± 13.8	0.543^†^

Data are presented as mean ± standard deviation or number (%) where applicable.

^a^Analysis was performed based on 46 patients (32 in the bevacizumab group and 14 in the observation group), excluding three patients in whom the presence of intraretinal hemorrhage was undeterminable.

^†^Statistical analysis was performed using Mann–Whitney U test.

^‡^Statistical analysis was performed using Fisher’s exact test.

^††^Statistical analysis was performed using chi-square test.

Both groups had similar characteristics, except for a greater extent of hemorrhage in the observation group than in the bevacizumab group (P = 0.039).

**Figure 2:**
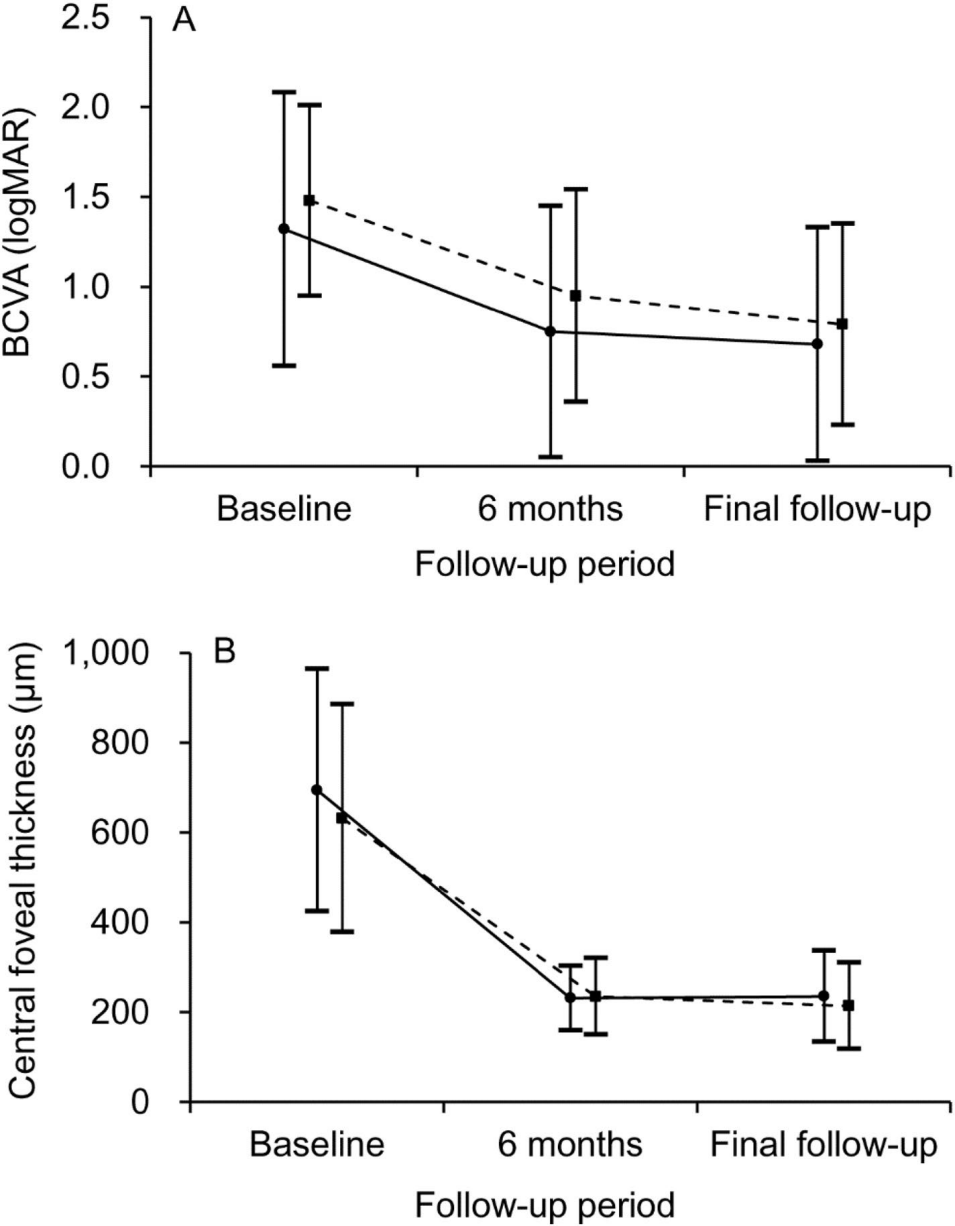
Changes in the BCVA (**A**) and CFT (**B**) in the two treatment groups. Solid lines (closed circle) indicate outcomes in patients treated with intravitreal bevacizumab, whereas dashed lines (solid square) indicate outcomes in patients who did not receive any treatment. There was no significant difference in the BCVA and CFT at diagnosis and the final follow-up between the two groups. Statistical analysis was performed using Mann–Whitney U test with Bonferroni correction. *BCVA* best-corrected visual acuity, *CFT* central foveal thickness, *LogMAR* logarithm of minimal angle of resolution.

Changes in the BCVA and CFT in the two groups are shown in Fig. 2.

In the bevacizumab group, the mean logMAR BCVA was 1.32 ± 0.76 at diagnosis, 0.75 ± 0.70 at 6 months, and 0.68 ± 0.65 at the final follow-up. The mean CFT was 631.6 ± 253.9 µm at diagnosis, 235.0 ± 85.2 µm at 6 months, and 213.9 ± 96.3 µm at the final follow-up. Lesion reactivation was noted in three (9.1%) patients, and ellipsoid zone disruption was noted in 13 (39.4%) patients at the final follow-up. In the observation group, the mean logMAR BCVA was 1.48 ± 0.53 at diagnosis, 0.95 ± 0.59 at 6 months, and 0.79 ± 0.56 at the final follow-up. The mean CFT was 693.9 ± 231.3 µm at diagnosis, 231.3 ± 71.9 µm at 6 months, and 235.4 ± 101.8 µm at the final follow-up. Lesion reactivation was noted in three (18.8%) patients, and ellipsoid zone disruption was noted in nine patients (56.3%) at the final follow-up. There was no significant difference in the BCVA at diagnosis (P = 0.724), the BCVA at final visit (P = 0.576), the CFT at diagnosis (P = 0.924), and the CFT at the final follow-up (P = 1.000) between the two groups. In addition, there was no significant difference in the incidence of lesion reactivation (P = 0.377) and ellipsoid zone disruption at the final visit between the two groups (P = 0.266).

Nineteen patients were included in the IRH group and 27 patients were included in the non-IRH group. Three patients in whom the presence of IRH was not accurately determined were excluded from this comparison. Table 3 summarizes the comparison of characteristics between the two groups.

**Table 3:** Comparisons of characteristics between the intraretinal hemorrhage group and non-intraretinal hemorrhage group.

Characteristics	IRH group (n = 19)	Non-IRH group (n = 27)	P-value
Age, years	77.8 ± 8.9	77.3 ± 7.8	0.858*
Male:female	7 (36.8%):12 (63.2%)	4 (14.8%):23 (85.2%)	0.159^†^
Diabetes mellitus	1 (5.3%)	2 (7.4%)	1.000^†^
Hypertension	13 (68.4%)	18 (66.7%)	0.901^‡^
Use of anticoagulants	4 (21.1%)	8 (29.6%)	0.735^†^
**Blood pressure, mmHg**			
Systolic	136.8 ± 14.9	141.4 ± 16.9	0.515*
Diastolic	75.5 ± 11.3	76.5 ± 12.2	0.946*
Extent of hemorrhage, disc areas	10.7 ± 5.2	7.1 ± 4.3	0.019*
Presence of submacular hemorrhage	16 (84.2%)	9 (33.3%)	0.001^‡^
**Treatment**			0.887^‡^
Intravitreal bevacizumab	13 (68.4%)	19 (70.4%)	
Observation	6 (31.6%)	8 (29.6%)	
Follow-up period, months	22.6 ± 14.9	26.3 ± 15.9	0.371*

Data are presented as mean ± standard deviation or number (%) where applicable.

*IRH* intraretinal hemorrhage.

*Statistical analysis was performed using Mann–Whitney U test.

^†^Statistical analysis was performed using Fisher’s exact test.

^‡^Statistical analysis was performed using chi-square test.

Both groups had similar characteristics, except for a greater extent of hemorrhage in the IRH group than in the non-IRH group (P = 0.019).

**Figure 3:**
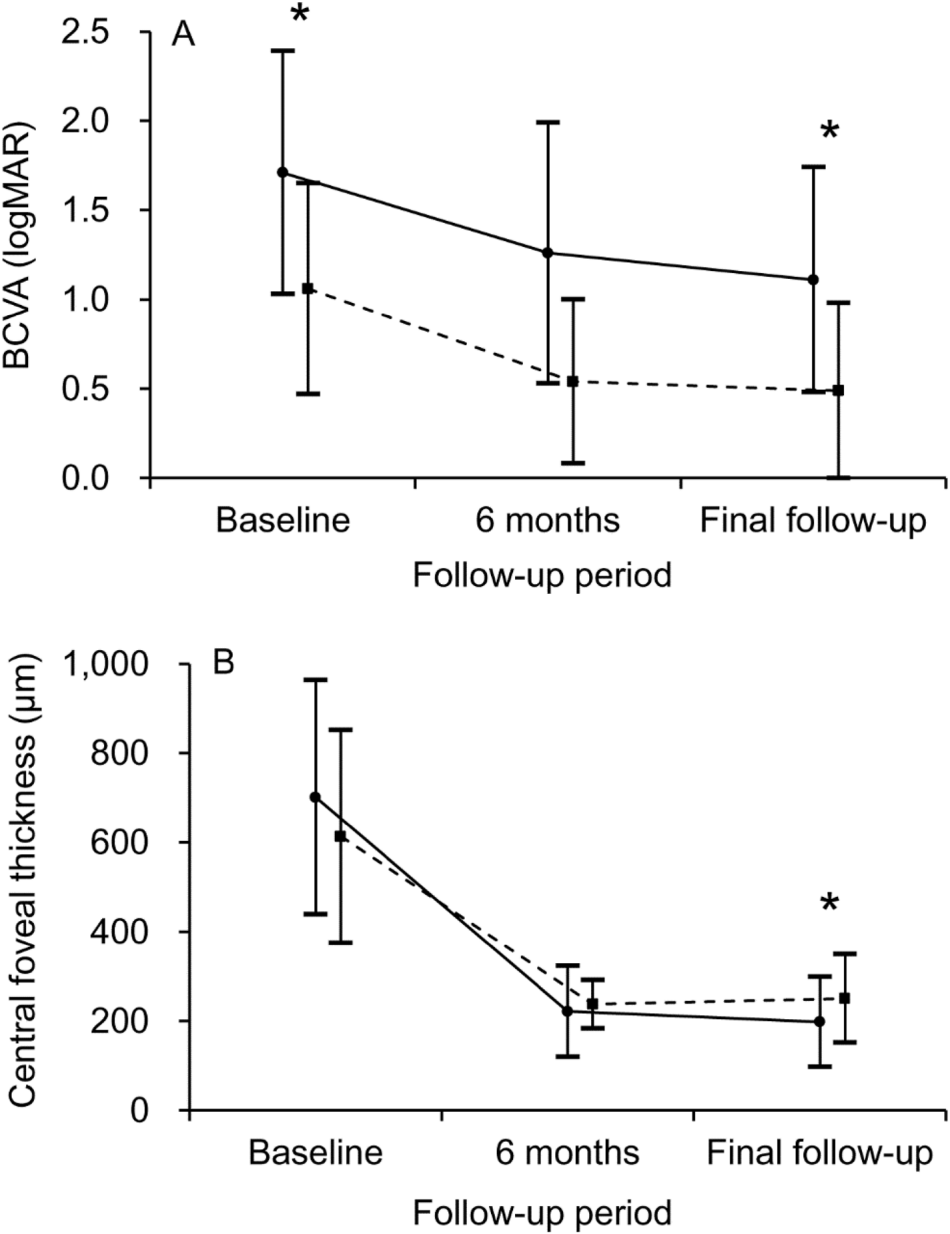
Changes in the BCVA (**A**) and CFT (**B**) in the IRH and non-IRH group. Solid lines (closed circle) indicate the IRH group and dashed lines (solid square) indicate the non-IRH group. The BCVA at baseline and the final follow-up were significantly worse in the IRH group than in the non-IRH group (**A**, asterisks). There was no difference in the central foveal thickness (CFT) at baseline; however, the CFT at the final follow-up was significantly lower in the IRH group than in the non-IRH group (**B**, asterisk). Statistical analysis was performed using Mann–Whitney *U* test with Bonferroni correction. *BCVA* best-corrected visual acuity, *LogMAR* logarithm of minimal angle of resolution, *IRH* intraretinal hemorrhage.

Changes in the BCVA and CFT in the two groups are shown in Fig. 3.

**Figure 4:**
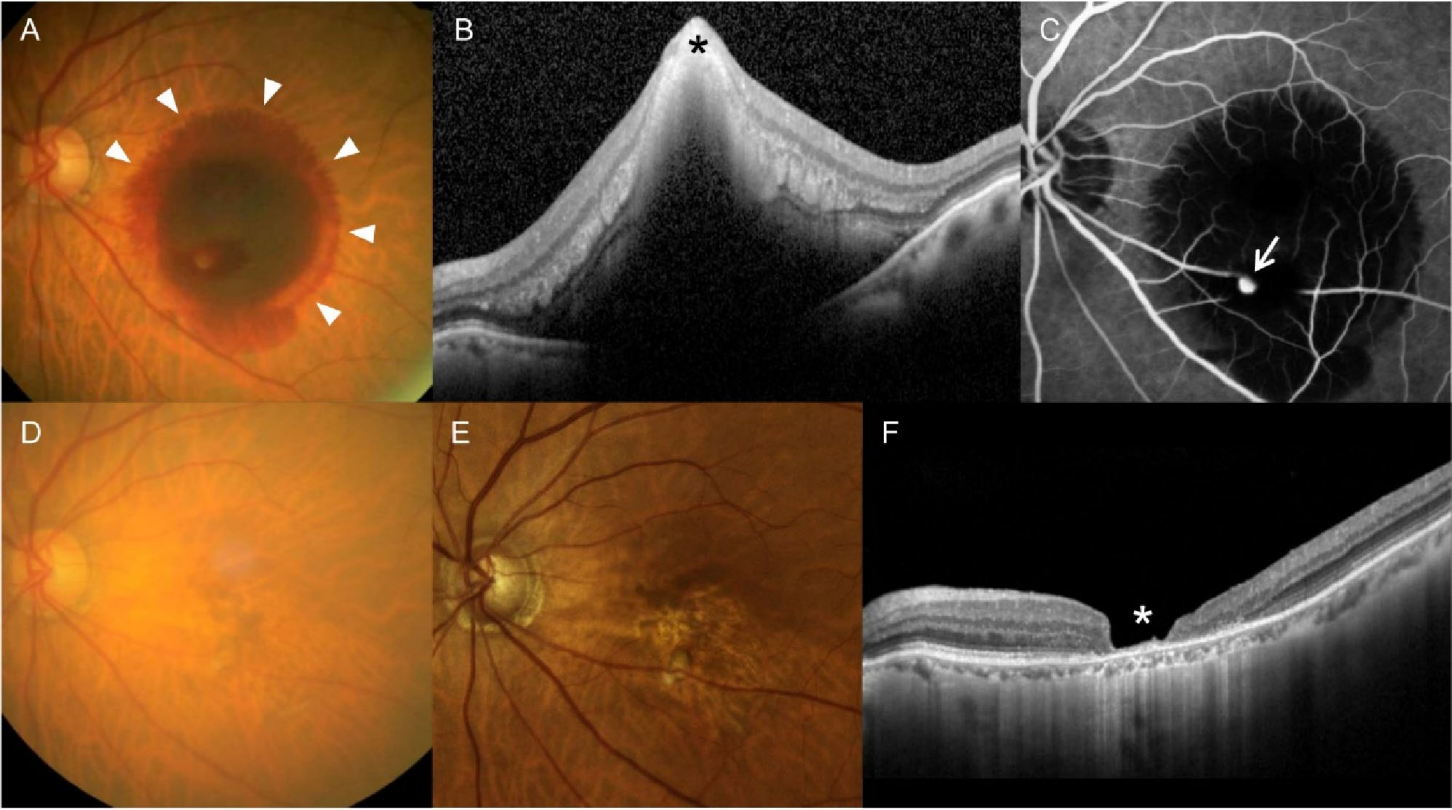
A representative case showing clinical outcomes in an 83-year-old patient diagnosed with hemorrhagic retinal arterial macroaneurysm (RAM). (**A**) At diagnosis, subretinal and intraretinal hemorrhage were noted, accompanied with fluffy hemorrhage (arrowheads). (**B**) An optical coherence tomography (OCT) image showing intraretinal hemorrhage at the fovea (black asterisk). A fluorescein angiography image showing a RAM lesion (**C**, arrow). The best-corrected visual acuity (BCVA) was measured as 20/1000. The patient was closely followed up without any treatment. (**D**) At 6 months, the hemorrhage was completely resolved. (**E**,**F**) At 15 months, there was no recurrence of hemorrhage or exudation. However, extreme retinal thinning at the foveal region was noted on OCT (**F**, white asterisk). The BCVA was measured as counting finger visual acuity.

In the IRH group, the mean logMAR BCVA was 1.71 ± 0.68 at diagnosis, 1.26 ± 0.73 at 6 months, and 1.11 ± 0.63 at the final follow-up. The mean CFT was 701.1 ± 262.2 µm at diagnosis, 221.7 ± 101.8 µm at 6 months, and 198.1 ± 101.2 µm at the final follow-up. Lesion reactivation was noted in two (10.5%) patients, and ellipsoid zone disruption was noted in 15 (78.9%) patients at the final follow-up. Fluffy hemorrhage was noted in nine patients. In the non-IRH group, the mean logMAR BCVA was 1.06 ± 0.59 at diagnosis, 0.54 ± 0.46 at 6 months, and 0.49 ± 0.49 at the final follow-up. The mean CFT was 613.3 ± 238.2 µm at diagnosis, 237.6 ± 54.1 µm at 6 months, and 250.6 ± 99.2 µm at the final follow-up. Lesion reactivation was noted in four (14.8%) patients, and ellipsoid zone disruption was noted in seven (25.9%) patients at the final follow-up. The BCVA at diagnosis (P = 0.006) and the BCVA at the final follow-up (P = 0.002) were significantly worse in the IRH group than in the non-IRH group. In addition, the CFT at the final follow-up was significantly lower in the IRH group than in the non-IRH group (P = 0.037). There was no difference in the CFT at diagnosis between the two groups (P = 0.384). There was no significant difference in the incidence of lesion reactivation (P = 1.000). The incidence of ellipsoid zone disruption at the final visit was significantly higher in the IRH group than in the non-IRH group (P < 0.001). Figure 4 shows the clinical course of a representative case of IRH.

In the multivariate analysis using IRH as a factor, older age (P = 0.036) and the presence of IRH (P = 0.007) were found to be associated with worse logMAR BCVA < 0.4 at the final follow-up (Table 4).

**Table 4:** Association between patients’ characteristics and visual acuity at the final follow-up using intraretinal hemorrhage as a factor (n = 46).

Morphologic features	P-value*	*β*	95% confidence interval
Age	0.036	1.193	1.012–1.406
Sex	0.766		
Diabetes mellitus	0.418		
Hypertension	0.273		
Use of anticoagulants	0.441		
Systolic blood pressure	0.335		
Diastolic blood pressure	0.958		
Extent of hemorrhage	0.664		
Intraretinal hemorrhage	0.007	29.417	2.538–341.015
Foveal involvement (hemorrhage vs. exudation)	0.987		
Treatment (intravitreal bevacizumab vs. observation)	0.306		

*Statistical analysis was performed using binary logistic regression.

In the multivariate analysis using submacular hemorrhage as a factor, older age (P = 0.044) and the presence of submacular hemorrhage (P = 0.041) were found to be associated with worse logMAR BCVA < 0.4 at the final follow-up (Table 5).

**Table 5:** Association between patients’ characteristics and visual acuity at the final follow-up using submacular hemorrhage as a factor (n = 46).

Morphologic features	P-value*	*β*	95% confidence interval
Age	0.044	1.159	1.004–1.339
Sex	0.260		
Diabetes mellitus	0.199		
Hypertension	0.155		
Use of anticoagulants	0.423		
Systolic blood pressure	0.483		
Diastolic blood pressure	0.865		
Extent of hemorrhage	0.552		
Submacular hemorrhage	0.041	9.671	1.041–89.822
Foveal involvement (hemorrhage vs. exudation)	0.841		
Treatment (intravitreal bevacizumab vs. observation)	0.788		

*Statistical analysis was performed using binary logistic regression.

When divided into two groups, according to the follow-up period, 23 patients were included in the long follow-up group (> 20 months) and 26 patients were included in the short follow-up group (< 20 months). In the long follow-up group, the mean logMAR BCVA was 1.39 ± 0.67 at diagnosis and 0.75 ± 0.55 at the final follow-up. In the short follow-up group, the values were 1.36 ± 0.74 at diagnosis and 0.68 ± 0.69 at the final follow-up, respectively. There was no difference in the BCVA at diagnosis (P = 0.991) and at the final follow-up (P = 0.333) between the two groups.

When the counting finger and hand motion visual acuities were converted to logMAR values 2 and 2.3, respectively, the mean logMAR BCVA was 1.28 ± 0.68 at diagnosis and 0.68 ± 0.65 at the final follow-up in the bevacizumab group. The values were 1.48 ± 0.53 at diagnosis and 0.79 ± 0.56 at the final follow-up in the observation group. There was no significant difference in the BCVA at diagnosis (P = 0.362) and the BCVA at final visit (P = 0.238) between the two groups. In the IRH group, the mean logMAR BCVA was 1.64 ± 0.56 at diagnosis and 1.11 ± 0.63 at the final follow-up. In the non-IRH group, the values were 1.06 ± 0.59 at diagnosis and 0.49 ± 0.49 at the final follow-up. The BCVA at diagnosis (P = 0.003) and at the final follow-up (P = 0.001) was significantly better in the non-IRH group than in the IRH group.

## Discussion

In our patients, the visual acuity significantly improved; three lines or greater visual improvement was noted in most patients. However, the visual improvement was limited in some patients with ellipsoid zone disruption at the fovea. The incidence of lesion reactivation was relatively low.

Treatment for RAM primarily focuses on subretinal hemorrhage and the RAM lesion itself. The blood has a negative impact on the retina, causing iron toxicity and mechanical damage^17,18^. Thus, management of subretinal hemorrhage using vitrectomy and tissue plasminogen activator injection, pneumatic displacement, or both have been considered useful therapeutic options for hemorrhagic RAM^2^. In addition, laser photocoagulation has been widely used to treat the RAM lesion^2^. After the advent of anti-VEGF agents, anti-VEGF therapy has also been used as the first-line treatment^2^ or an adjunctive to laser photocoagulation^19^. Although these treatment methods are effective, there is no gold-standard treatment for RAM; many doctors also recommend close observation without treatment^2^.

In our study, approximately two-thirds of the patients were treated with intravitreal bevacizumab. Anti-VEGF therapy for RAM was first introduced to control exudation from the aneurysm^20,21^. In a study by Chanana and Azad, a marked decrease in edema was observed after bevacizumab injection^20^. This favorable outcome was also reported by a study by Jonas and Schmidbauer^21^. After these preliminary studies, researchers have demonstrated that anti-VEGF therapy is also effective in hemorrhagic cases. Javey et al. were the first to report on outcomes of bevacizumab in hemorrhagic RAM^22^. In their report, marked improvement in visual acuity was noted with a hemorrhage resolution after bevacizumab injection^22^. Pichi et al. analyzed the efficacy of bevacizumab therapy in 37 patients, of which 19 had hemorrhagic RAM^13^. In their study, a significant anatomical and functional improvement was noted during the 6-week follow-up period. In addition, there was no notable difference in treatment outcomes between hemorrhagic RAM and exudative RAM. In a study by Cho et al., faster visual recovery was noted in patients treated with bevacizumab than those who were not^23^. A recent study by Mansour et al. also showed that anti-VEGF therapy is effective for the treatment of RAM^24^.

Although the lack of prospective case–control clinical trials limits the solid conclusion regarding its efficacy, previous studies have suggested that anti-VEGF therapy has some valid effect in RAM treatment. However, the influence of initial bevacizumab therapy on the long-term outcomes of hemorrhagic RAM is unclear.

In our study, we compared the long-term outcomes of hemorrhagic RAM (mean 24.8 months) between patients with and without bevacizumab therapy. The visual and anatomical outcomes were similar between both groups, suggesting that bevacizumab therapy may not significantly influence the long-term outcomes. There were no strict guidelines on the use of bevacizumab in the present study. There was no significant difference between the bevacizumab group and the observation group, except for the hypertension. However, the lack of selection guideline in the study result should be kept in mind.

In this study, the influence of IRH at the fovea on the long-term outcomes was also evaluated. Patients with IRH showed worse visual outcomes with a higher incidence of ellipsoid zone disruption than those without IRH. In addition, in multivariate analysis, the presence of IRH was significantly associated with poor visual outcomes. Doi et al. recently investigated the influence of macular IRH on short-term visual outcomes of hemorrhagic RAM^16^. In their study, 23 patients were treated using vitrectomy, subretinal injection of tissue plasminogen activator, and air tamponade. The final visual outcome was measured at 6 months; postoperative visual acuity was significantly worse in patients with IRH than patients without IRH. Doi et al. have suggested that the presence of both subretinal hemorrhage and IRH may accelerate photoreceptor damage by exposing the photoreceptor cells to the blood from both sides of the subretinal space and the outer plexiform layer^16^. In our study, the incidence of submacular hemorrhage was higher in patients with IRH than those without IRH, suggesting that the presence of submacular hemorrhage may influence the poor visual outcome in patients with IRH. Furthermore, both IRH and submacular hemorrhage were found to be closely associated with poor visual outcome.

In our study, patients were treated with intravitreal bevacizumab or did not receive any treatment. In addition, our study was based on longer follow-up data with a larger study population. Despite these differences, our study result—significantly worse anatomical and functional outcomes in patients with IRH than in patients without IRH—was consistent with that reported by Doi et al.^16^ This may highlight the negative influence of IRH on the prognosis of hemorrhagic RAM.

Fluffy hemorrhage, which represents IRH spreading radially^16^, was noted in approximately half of our patients in the IRH group. In the study by Doi et al., the incidence of fluffy hemorrhage was 64.7% among the patients with macular IRH^16^. A similar shape of IRH was previously reported in neovascular age-related macular degeneration, especially in polypoidal choroidal vasculopathy^25^. To date, only a few clinical studies have evaluated the nature and clinical significance of this peculiar type of IRH^16,25^. Further histopathologic studies are needed to better elucidate its origin and impact on adjacent retinal tissue.

Previous reports have shown that recurrent hemorrhage can develop after one successful RAM treatment^26^. However, the long-term incidence of lesion reactivation in hemorrhagic RAM is unclear. In our study, lesion reactivation was noted as 12.2% patients during the mean follow-up of 24.8 month. Although the incidence was not high, most events occurred within the first 6 months, suggesting the need for close observation during this period.

In addition to its retrospective nature, this study has some limitations. First, this study was not controlled. There was no strict guideline on the use of bevacizumab. Moreover, the use of bevacizumab was not randomly assigned to patients. Second, this study included only hemorrhagic RAM cases that involved the fovea. Thus, our results may not be valid for exudative RAM or cases without foveal involvement. Third, all the patients were treated with intravitreal bevacizumab or observed without treatment. Thus, our results do not reflect outcomes in patients who underwent other treatments, such as surgical displacement of hemorrhage, laser photocoagulation, or anti-VEGF therapy using ranibizumab or aflibercept. Fourth, there was a large variation in the follow-up period among the patients. However, since the visual outcomes were comparable between the long and the short follow-up groups, we believe that the variation in the follow-up period may not significantly influence the study results. Lastly, in multivariate analysis, the cutoff value of logMAR 0.4 was arbitrarily determined.

In summary, during the mean follow-up of 24.8 months, a significant visual and anatomical improvement was noted in hemorrhagic RAM. In our patients, the presence of IRH at the fovea was associated with poor long-term outcomes, whereas bevacizumab therapy did not significantly influence the outcomes. Considering the retrospective nature of the present study, however, further experimental studies are required to reveal the influence of IRH more accurately and the effect of bevacizumab therapy on vision prognosis.

## Data Availability

The datasets generated during and/or analyzed during the current study are available from the corresponding author upon reasonable request.
